# Effects of organic fertilizers via quick artificial decomposition on crop growth

**DOI:** 10.1038/s41598-021-83576-4

**Published:** 2021-02-16

**Authors:** Xuemiao Ma, Haixiao Li, Yan Xu, Cunshou Liu

**Affiliations:** 1 College of Natural Resources and Environment, Northwest A&F University, Yangling, 712100 Shaanxi China; 2 College of Environment Science and Engineering, Nankai University, Tianjin, 300350 China; 3 Tianjin Key Laboratory of Environmental Technology for Complex Trans-Media Pollution, Tianjin, 300350 China; 4 Department of Soils and Agri-Food Engineering, Paul Comtois Bldg, Laval University, Quebec, QC G1K 7P4 Canada

**Keywords:** Plant sciences, Environmental sciences, Energy science and technology

## Abstract

Applying organic matters into the soil would help to improve soil quality and sustain crop production. In addition, the small molecular organic matters could be active in influencing soil nutrient cycling and crop development. Thus, this study has firstly induced a new technology of quick artificial decomposition to produce fertilizers containing small molecular organic compounds from crop residues and other biological wastes. The fertilizers were produced via the quick artificial decomposition from biological wastes. The small organic species in the fertilizers were identified by the LC–MS. Field experiments of kiwifruit were conducted to test the effects of fertilizers. In total, 341 species of small organic matters have been determined in the produced fertilizers. The results showed that the organic fertilizers could significantly increase the yields of kiwifruit by 15.2% in contrast with mineral fertilizer treatments. Whereas, the organic fertilizers could enhance the contents of nutritive components in kiwifruits. These results proved that the organic fertilizers containing more small organic matter could be more efficient in promoting crop production.

## Introduction

Mineral fertilizers are important to food security for the world. World fertilizer consumption steadily increased at 1.6 percent per annum from 2015 to 2019 according to the world fertilizer outlook to 2019 of Food and Agriculture Organization of the United Nations (FAO). However, the use of chemical fertilizers might cause several soil problems such as soil compaction and degradation^1^. Increasing soil organic matter (SOM) content is commonly recommended as an efficient way to improve soil quality for sustainable agricultural production^2,3^. SOMs help to improve soil quality and crop production via various functions such as creating more macro-aggregates for soil stability^4^, favoring the activities of soil microorganisms with more carbon sources^5^, and enhancing nutritive element availability for crop uptake^6,7^.

Conventional methods to increase SOM contents includes straw returning and composting. However, those methods have several defects in providing organic matter into soil. Firstly, during the process of straw returning or composting, the added bio-materials would partly decompose and transform into other forms of organic matters before they could be effective to improve soil quality. That decomposition process would lead to the emissions of greenhouse effect gases and carbon losses. It has been mentioned that different composting operations could cause a range of CH_4_ emissions, accounting for 0.01–8% of the total carbon loss regardless of the biogenic CO_2_ emissions^8^. Whereas Ma et al. (2008) have found that the wheat straw returning would significantly increase almost twice the total CH_4_ emission in a field of paddy soil^9^. Moreover, methods of straw returning and composting would induce large amounts of great molecular weight organic matters via the polycondensation during the humification^10^. While other theory excluding “humification” thought that the bio-materials would decompose into large biopolymers^11^. Although great molecular weight organic matters are important for soil health, small molecular organic matters could be more active in influencing soil nutrient cycling and crop development. Low molecular organic matters such as low molecular organic acids would contribute significantly to increase the availability of soil nutrients^12,13^. It is also believed that the small organic matters are more assimilable than large organic molecules^11^. While some small molecular organic compounds (e.g. amino acids, organic–metallic complexes, and sugar) could even be directly taken and used by crops^14–16^. It could be reasonable that the direct absorbed small organic matters could be used more efficiently by crops without some assimilation processes.

In this context, we induced a new technology of quick artificial decomposition to produce organic fertilizers containing small molecular weight organic matters from crop residues and other biological wastes. That process carried on in closed reactors to avoid carbon losses. The product compositions were determined at first, and its effects on crop growth were tested by a cash crop (kiwifruit, *Actinidia chinensis*) trial in comparison with mineral fertilizers and conventional organic fertilizers.

## Materials and methods

### Fertilizer production via quick artificial decomposition

Raw materials including straw, dregs, sludge, manure, weathered coal, etc. can be used to produce fertilizers via the quick artificial decomposition. The technology uses a small number of FeSO_4_ (0.3–3%, m/m of raw materials) to guide the reaction to degradation development. It uses TiO_2_ (3–12.5%, m/m of raw materials) as oxidizing agents and KOH (2.5–20%, m/m of raw materials) as hydrolyzing agent for lignin oxidation^17^ and hydrolysis of protein and cellulose^18,19^. Meanwhile, to provide a favorable metabolic environment for the reaction, the conditions of temperature and pressure are set at 80–175 ℃ and 3–12.5 atmospheres, respectively. Raw materials are firstly screened and broken before entering the reactor. Then the pretreated materials are degraded and activated in the reactor for 40 min, and this step is followed by a decompressing blasting. After that, a concentrating process is conducted to form the final products of fertilizer.

The product quality of the fertilizers is controlled by the measurements set in the agriculture industry standard of the People's Republic of China (NYT 1971–2010, NYT 1976–2010) for water-soluble fertilizers containing humic-acids, including the analysis of nutritive elements, metallic elements, humic substance contents and soluble organic matter contents. The species of small molecular organic matters in the fertilizers were identified by the method of LC–MS using the RRLC-Q-TOF 1200 rapid separation liquid chromatograph and the 6520-quadrupole mass spectrometer of Agilent Technologies Inc. (USA). The measurement conditions were set as follows: (1) For the liquid chromatograph system, the mobile phase A was 0.1% formic acid and the mobile phase B was the acetonitrile containing 0.1% formic acid. The analytical column was Agilent SB-C18 column with a dimension of 3.0 mm × 100 mm and the particle size at 1.8 μm. The liquid flow rate was 0.3 mL min^-1^. For the separation, mobile phase B remained 2% in 0–3 min. Phase B rose from 2 to 10%, from 10 to 50%, and from 50 to 90% in 3–10 min, 10–30 min, and 30–40 min, respectively. Then, phase B content decreased from 90 to 2% in 40–41 min and remained 2% in 41–45 min. The time for the post-column equilibrium was 8 min. The injection volume for the sample was 5 μL. (2) For the mass spectrometry, the ion source was + ESI. The determination was conducted in both positive and negative ion modes. The quadrupole temperature was set at 100 ℃. The capillary voltage was 3500 V and the voltage for the fragmentor was 175 V. The atomizing pressure was 35 psig. The drying gas temperature was 325 ℃ while the drying gas flow was 10 L min^-1^. The mass scanning range was set within 50–1100 m/z.

### Field experiments

The experimental site is located in Jiaofang Village, Jinqu Town, Mei County, Shaanxi Province. The basic physical and chemical soil properties of the orchard are: soil pH 7.55, organic matter 13.98 g kg^-1^, alkali nitrogen 84.80 mg kg^-1^, available phosphorus 38.13 mg kg^-1^, available potassium 177.73 mg kg^-1^, available iron 6.83 mg kg^-1^. The kiwifruit variety is "Hayward"; and the trees were planted in 2004 with row spacing at 3 m and plant spacing at 2 m. The fertilization experiment on kiwifruits lasted for three years from 2011 to 2013. Three sorts of fertilizers had been used in this experience, including: 1. Artificial decomposition produced organic fertilizer (N 20%, P_2_O_5_ 10%, K_2_O 12%, water-soluble organic matter 35%, and mineral elements 8%); 2. A mixture of mineral fertilizers as urea (N 46%), superphosphate (P_2_O_5_ 16%) and potassium sulfate (K_2_O 50%); 3. Mixture of mineral fertilizers and conventional organic fertilizer of pig manure (N 0.5%, P_2_O_5_ 0.54%, K_2_O 0.42%, and water-soluble organic matter 15%).

It was a complete random design for this experiment with three fertilizer treatment: 1. Artificial decomposition produced organic fertilizer at 2.0 kg per each plant (equivalent to 0.4 kg N, 0.2 kg P_2_O_5_ and 0.24 kg K_2_O per plant); 2. Conventional organic fertilizer plus mineral fertilizers with 32 kg fermented pig manure, 0.52 kg urea, 0.18 kg superphosphate, and 0.22 kg potassium sulfate per plant (same amounts of N, P, K as artificial decomposition produced organic fertilizer treatment); 3. Mineral fertilizers of 0.87 kg urea, 1.25 kg superphosphate, and 0.48 kg potassium sulfate. These treatments are represented as AF, OF + MF and MF. During the season, the fertilizers were applied in three times as base fertilizer, pre-flowering fertilizer and swelling fertilizer, referring to 30%, 40% and 30% of the total amount. In the orchard, 15 continuous trees were counted as a plot separated by another three trees from the next plot. Each fertilizer treatment was repeated three times in three plots randomly.

The annual yields were measured for each plot as well as the average fruit weight during the three-year experiment. 30 fruits were sampled in each plot for the measurement of Brix value using refractometer and firmness using firmness tester. In addition, the acidity, the vitamin C content, and the concentrations of P, K, Ca and Zn were determined by the Testing Center of Northwest A&F University through the methods of pH-3C meter, liquid chromatography (LC-10A, Shimadzu Corporation, Japan), and ICP-AES (SPS8000, China), respectively.

The soil samples in each plot have been collected every year during the flowering and ripening periods of kiwifruit plants. The total carbon and total nitrogen contents of soil samples were analyzed by the element analyzer (Vario MACRO cube, Elementar, Germany). The available soil phosphorus was extracted by Olsen extraction^20^ and determined by Murphy Riley method^21^, while the available soil potassium was extracted with 1 M NH_4_OAc solution and determined by atomic absorption spectrophotometry (PinAAcle 900F, PerkinElmer). The soil organic matter content was determined with the standard method described in GB 9834-88.

### Data analysis

The ANOVA of the experimental data was conducted by the software SAS with the MIXED command to determine the effect of fertilizers on selected variables. The effect of fertilizer was deemed as the fixed effect, while there was no random effect. The factor of year was regarded as the repeated measurement for the data analysis. The effect was deemed as significant at a level of α = 0.05. For the multiple comparisons, the Duncan test was used to identify the significant difference (*P* < 0.05) between fertilizer treatments on selected variables.

## Results

### Organic matter species in produced fertilizers

The method of modified Fenton reaction was hundreds of folds faster than microorganism decomposition. The raw materials were converted into small molecular soluble organic matters within four hours, while there was only 0.0012% of C losses. Depending on the derived bio-material sources, the average water-soluble organic matter content and average humic substance content were 35% and 7% (m/m). In total, 343 species of organic matters have been determined by the LC–MS (Table A.1) including various organic acids, esters, alcohols, pyrimidines, saccharides, and small peptides. The average molecular mass of those organic matters was 288.67. More than half of the species (57.30%) had a molecular mass smaller than 300, while only 4.97% of the organic matters had a molecular mass over 500.

### Fertilizer effects on kiwifruit

AF treatment could significantly increase several mineral contents (Ca and N) of kiwifruits in contrast with MF and MF + OF treatments (Table 1). However, although for the elements of K, P, and Zn there was no significant difference observed within the treatments, AF treatment had relatively higher values. AF treatment significantly enhances kiwifruit production with a 15.2% higher yield than that of MF (Table 2).

**Table 1:** Effects of different fertilizer treatments on nutritive element contents of kiwifruit.

Treatments	Element content (DW) (g kg^-1^)				
	N	P	K	Ca	Zn
MF	14.3 ± 0.84b	10.5 ± 0.78a	143.7 ± 7.90a	38.9 ± 7.63b	1.19 ± 0.20a
MF + OF	14.8 ± 0.18b	10.7 ± 0.28a	145.4 ± 11.24a	40.5 ± 9.24ab	1.20 ± 0.16a
AF	15.9 ± 0.47a	11.2 ± 0.20a	150.8 ± 12.00a	46.6 ± 4.77a	1.23 ± 0.10a

The different letters after the values indicated the significant differences (P < 0.05) of kiwifruit nutritive element contents between the treatments. The standard errors (n = 3) are presented.

**Table 2:** Effects of fertilizer treatments on yield per tree of kiwifruit.

Treatments	Yield per tree (kg)	Increase rate (%)
MF	18.7 ± 2.73b	–
MF + OF	19.6 ± 2.10ab	4.8
AF	21.5 ± 1.26a	15.2

The different letters after the values indicated the significant differences (P < 0.05) of kiwifruit yields between the treatments. The standard errors (n = 3) are presented.

**Figure 1:**
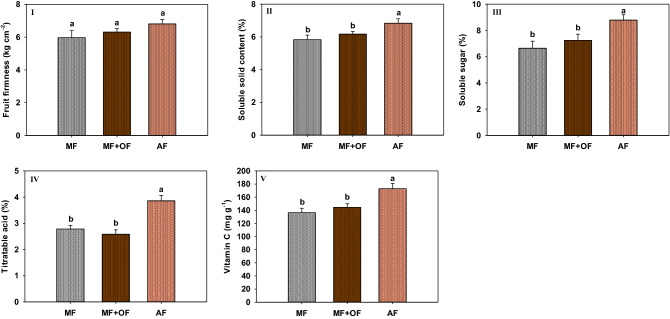
The fruit firmness (kg cm^-2^) (**I**), the soluble solid content (%) (**II**), the soluble sugar content (%) (**III**), the titratable acid content (%) (**IV**), and Vitamin C content (mg per 100 g) (**V**) of kiwifruits under the treatments of AF (Fenton reaction produced fertilizer at 2.0 kg per each plant, equivalent to 0.4 kg N, 0.2 kg P2O5 and 0.24 kg K2O per plant), OF + MF (organic fertilizer plus mineral fertilizers with 32 kg fermented pig manure, 0.52 kg urea, 0.18 kg superphosphate, and 0.22 kg potassium sulfate per plant), and MF (mineral fertilizers of 0.87 kg urea, 1.25 kg superphosphate, and 0.48 kg potassium sulfate). The different letters in each sub-figure indicated the significant differences of the variable between the samples under different treatments. The vertical bars represent the standard errors for the variable values.

Besides, AF also profited fruit quality. Shown in Fig. 1, fruits under AF treatment had relatively higher firmness and significantly higher soluble solid, soluble sugar, titratable acid and vitamin C contents than the other treatments.

### Fertilizer effects on soil nutrients

The results of fertilizer effects on soil nutrients are presented in Table 3. Across the fertilization regimes, soils had smaller average values of alkali nitrogen and available phosphorus at ripening period than those at flowering period. In contrast, the average available potassium and organic matter contents in soils were relatively higher at ripening than those at flowering. While the total carbon in soil maintained at a similar level from flowering to ripening.

**Table 3:** Effects of different fertilization treatments on soil nutrients of kiwifruit culture.

Period	Treatment	Alkali nitrogen g kg^-1^	Total carbon g kg^-1^	Available phosphorus mg kg^-1^	Available potassium mg kg^-1^	Total organic matters g kg^-1^
-	Basic soil	0.08 ± 0.01	-	38.13 ± 3.50	177.73 ± 8.26	13.98 ± 0.09
Flowering	MF	0.24 ± 0.01b	2.08 ± 0.11ab	74.50 ± 0.26ab	304.07 ± 7.75ab	13.50 ± 0.08b
	MF + OF	0.37 ± 0.01a	3.15 ± 0.09b	123.40 ± 4.86b	455.97 ± 9.02b	18.00 ± 0.65a
	FF	0.33 ± 0.03a	5.25 ± 0.53a	136.77 ± 17a	590.2 ± 128a	19.00 ± 0.57a
Ripening	MF	0.28 ± 0.02b	2.29 ± 0.20b	64.87 ± 2.76b	509.73 ± 39.05b	14.60 ± 0.24b
	MF + OF	0.43 ± 0.03a	4.03 ± 0.24a	108.33 ± 5.31a	521.23 ± 22.66b	25.70 ± 0.28a
	FF	0.30 ± 0.02a	4.64 ± 0.01a	100.27 ± 4.32a	625.23 ± 8.01a	24.00 ± 0.07a

The different letters after the values indicated the significant differences (P < 0.05) of soil nutrients between the treatments. The standard errors (n = 9) are presented.

In terms of the fertilizer effects, soil under AF treatment had significantly higher values of almost all the analyzed variables (total carbon, available phosphorus, available potassium, and organic matter content) followed by the values under MF + OF treatment. The MF treatment led to the significantly lower level of soil nutrients at both flowering and ripening periods.

## Discussion

### Organic fertilizer production

The plants litter would be decomposed and mineralized in soil through the activities of microorganisms, while the recalcitrant aromatic compounds in plant residues and microbial biopolymers would be preserved^22^. The direct transformation of plant litter and microbial residues as well as the microbial resynthesis of the small organic matters could contribute to the accumulation of humic substances in soil. Under natural conditions, that humification through microbial activities is a relatively slow process. According to Tavares et Nahas (2014)^23^, after a 30-day incubation the humification rate in the soils of the forest, pasture, and maize crop field were 34.21%, 60.34%, and 94.96%, respectively. In contrast, the quick artificial decomposition in this study could accelerate the plant residue decomposition process in 40 min. Since it was a chemical hydrolyzing and oxidizing process, the reaction would convert the raw materials of plant residues more thoroughly into small molecular organic matters. Thus, compared to the decomposition by microorganism, the humification degree of the produced fertilizers was relatively small at about 20% (calculated from 7% humic substance contents and 35% total soluble organic matter contents in the fertilizers), while the humification degree in soils were generally over 50%^23,24^. In terms of the small molecule organic matters, 343 species of organic matters with a molecular mass inferior to 500 daltons were determined in the produced fertilizers. Compared to the passive SOMs such as the humic substances with a great molecular mass over thousands of daltons^25^, those organic matters would be probably considered as active organic matters as they might tend to be readily digestible and easily decomposed. In general, the active organic matters are only a small part (5–20%) of the total soil organic matters^26^. However, they play an important role in crop production due to the rapidly cycles nutrients for the feed of crops and microbes and the function of crop disease suppression^26,27^. The increased yields and improved fruit quality of kiwifruits under AF treatments have proven the positive effects of those small organic matters on crop growth.

**Figure 2:**
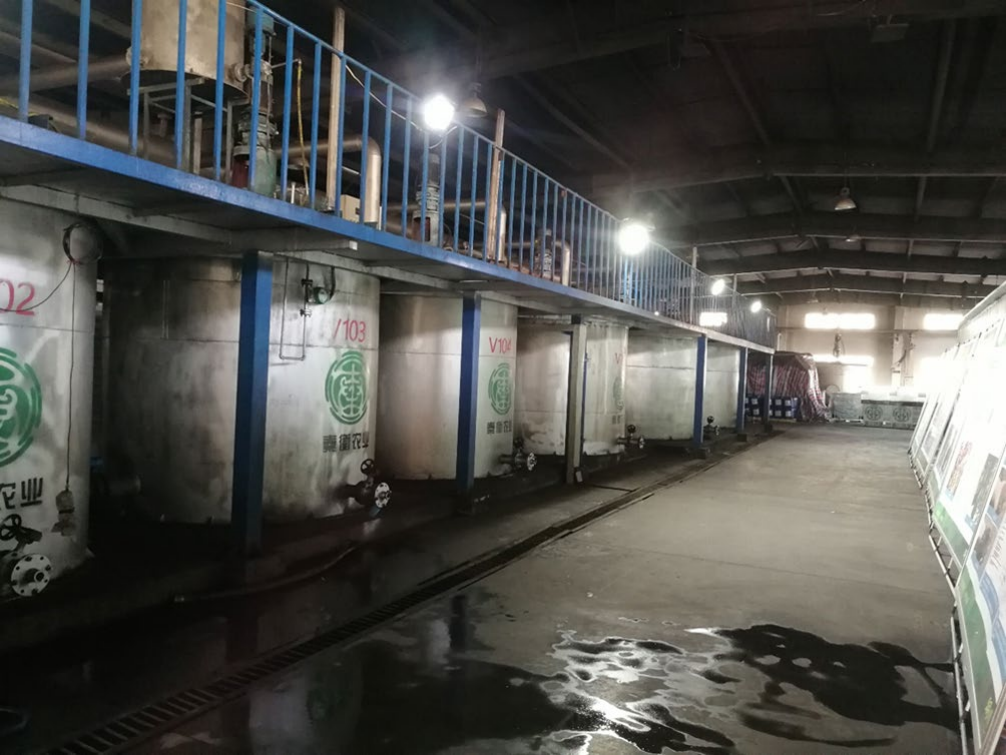
The reactors of the modified Fenton reaction equipment at the experimental site of Tongchuan, Shannxi, China.

On the other hand, decomposition and humification by microorganisms would cause CO_2_ and CH_4_ emission leading to the loss of C. Larionova al. (2017)^28^ reported that the C loss caused by the CO_2_ emission during the leaf decomposition and humification could attain to 20–80% of the total organic carbon depending on soil moistures. The whole procedure of the artificial decomposition was carried out in closed reactors (Fig. 2) so that the measured C loss was only 0.0012% with almost all organic carbon recycled.

### Organic fertilizer effects on plant production and soil nutrients

The application of the organic fertilizers improved the production of kiwifruits (Table 2). The effects of the produced organic fertilizer were more evident than that of the conventional organic fertilizer of fermented pig manure probably due to the existence of active small molecular organic matters. During the growth stage (flowering) of kiwifruit, more soil available phosphorus and available potassium were observed under AF treatment (Table 3). Firstly, small organic molecules are easier to be mineralized and release mineral nutrients into the soil. Small organic molecules such as low molecular weight organic acids have also been widely proved to contribute to soil P mobilizing via dissolution and complexation^29–31^. The formation of organic–metallic complexes via small molecular organic acids could enhance the availability of metallic elements^32^. It explained why significantly higher content of Ca was observed in the fruits of kiwifruit under AF treatment (Table 1).

Moreover, the AF application also profited fruit quality with higher firmness and higher soluble solid, soluble sugar, titratable acid and vitamin C contents (Fig. 1). Similar results had been reported in other studies. Yaping et al. (2017)^33^ have found organic matter content in soil was positively correlated with mass per fruit, soluble solids content, soluble sugar content and VC content for the fruit of jujuba. While Mauromicale et al. (2011)^34^ also reported that soil organic supplementation could significantly increase reducing sugar, soluble solid, titratable acidity, and ascorbic acid contents in tomato. Although the same amount of organic matter has been added by fermented pig manure, the production and the quality of kiwifruits were not improved under the MF + OF treatment. As Fallahi (2006)^35^ found that humic substances might not influence apple quality, it indicated that the recalcitrant and high molecular weight organic matters could not promote crop growth evidently.

### Roles of small molecular organic matters for crops

The theory of mineral plant nutrition has contributed to guarantee our food security since it was first induced by Justus von Liebig in 1840. But it should not be ignored that the growth of crops is the process of accumulating organic matter. Therefore, the most important element for crop growth is carbon. To some degree, the crops are in short of carbon uptake. For example, the enrichment of CO_2_ in the greenhouse can enhance crop production by supplying sufficient CO_2_ for photosynthesis^36,37^. Besides, crops could also take small organic matters through roots or leaves to support their growth. Sulmon et al. (2012)^38^ used exogenous carbohydrates to improve the phytoremediation, as plants could assimilate the exogenous carbohydrates.

In addition to carbon, plants might possess an innate ability to acquire N from organic sources directly without the prior microbial mineralization^15^. Hirner et al. (2006)^39^ have identified a high-affinity transporter for cellular amino acid uptake in the root epidermis. Ge et al. (2008)^15^ also reported that the uptake of glycine-N contributed 21% of N uptake for tomatoes after those of KNO_3_-N and NH_4_Cl-N. Other small N-molecules, such as urea, polyamines, and small polypeptides derived from enzymatic cleavage, could also be directly taken by crops^40^.

Comparing to taking CO_2_, taking and using directly the small molecular organic matters (e.g., amino acids, peptides, and carbohydrates) would be a more efficient way to promote crop growth without several assimilation steps. Moreover, crops would reallocate their organic matters to fight against the pressure of the environment^41,42^. Applying the active organic matters into soils might contribute to saving crops’ investments of C to counteract environment pressures.

This study has demonstrated that the application of organic fertilizers rich in active small molecular organic matters had the advantages in improving crop production and quality when compared to conventional organic fertilizers. However, there are still uncertainties for small molecular organic matters’ role for crops, such as their uptake pathway and fate inside crops. By clarifying those uncertainties, a compound understanding of small molecular organic matters would contribute to a more efficient way to improve crop production.

## Conclusion

Crops could directly uptake and use organic nutrients from soils. Organic matters should have small molecules to be efficiently used by crops such as amino acids, peptides, sugar and organic–metallic complexes. The new technology of quick artificial decomposition could efficiently convert biological wastes into organic fertilizer containing various small molecular organic matters to supply crops. Our studies had shown that the produced fertilizers could both increase crop yields and quality compared with mineral fertilizers and conventional organic fertilizers. It is reasonable to think that further studies on both fertilizer production improvement and different organic nutrient functions on crops would contribute to producing more efficient organic fertilizers.

## Supplementary Information

Supplementary Information.

## References

[1] Massah J, Azadegan B (2016). Effect of chemical fertilizers on soil compaction and degradation. AMA Agric. Mech. Asia Afr. Latin Am..

[2] Fageriaa NK (2012). Role of soil organic matter in maintaining sustainability of cropping systems. Commun. Soil Sci. Plant Anal..

[3] Liu XB, Zhang XY, Wang YX, Sui YY, Ding G (2010). Soil degradation: a problem threatening the sustainable development of agriculture in Northeast China. Plant Soil Environ..

[4] Peng X, Yan X, Zhou H, Zhang YZ, Sun H (2015). Assessing the contributions of sesquioxides and soil organic matter to aggregation in an Ultisol under long-term fertilization. Soil Tillage Res..

[5] Yang Y, Zhang N, Xue M, Lu ST, Tao S (2011). Effects of soil organic matter on the development of the microbial polycyclic aromatic hydrocarbons (PAHs) degradation potentials. Environ. Pollut..

[6] Gerke J (2010). Humic (organic matter)-Al(Fe)-phosphate complexes: an underestimated phosphate form in soils and source of plant-available phosphate. Soil Sci..

[7] Ch’ng HY, Ahmed OH, Majid NMA (2014). Improving phosphorus availability in an acid soil using organic amendments produced from agroindustrial wastes. Sci. World J..

[8] Ahn HK, Mulbry W, White JW, Kondrad SL (2011). Pile mixing increases greenhouse gas emissions during composting of dairy manure. Biores. Technol..

[9] Ma J, Xu H, Yagi K, Cai Z (2008). Methane emission from paddy soils as affected by wheat straw returning mode. Plant Soil.

[10] Wei Z (2014). Assessment of humification degree of dissolved organic matter from different composts using fluorescence spectroscopy technology. Chemosphere.

[11] Lehmann J, Kleber M (2015). The contentious nature of soil organic matter. Nature.

[12] Jha Y, Subramanian RB, Meena VS, Maurya BR, Verma JP, Meena RS (2016). Regulation of plant physiology and antioxidant enzymes for alleviating salinity stress by potassium-mobilizing bacteria. Potassium Solubilizing Microorganisms for Sustainable Agriculture.

[13] Mihoub A, Bouhoun MD, Naeem A (2018). Short-term effects of phosphate fertilizer enriched with low molecular weight organic acids on phosphorus release kinetic and availability under calcareous conditions in arid region. J. Sci. Agric..

[14] El-Naggar A, El-Araby A, Neergaard AD, Høgh-Jensen H (2008). Crop responses to 15 N-labelled organic and inorganic nitrogen sources. Nutr. Cycl. Agroecosyst..

[15] Ge T (2008). Amino acids as a nitrogen source for tomato seedlings: the use of dual-labeled (13 C, 15 N) glycine to test for direct uptake by tomato seedlings. Environ. Exp. Bot..

[16] Wickstrom, K. H. & Metzger, M. S. field application of sugars to increase crop yield. (2016).

[17] Dai J, Patti AF, Saito K (2016). Recent developments in chemical degradation of lignin: catalytic oxidation and ionic liquids. Tetrahedron Lett..

[18] Chi X (2019). A clean and effective potassium hydroxide pretreatment of corncob residue for the enhancement of enzymatic hydrolysis at high solids loading. RSC Adv..

[19] Paixão SM (2016). Sugarcane bagasse delignification with potassium hydroxide for enhanced enzymatic hydrolysis. RSC Adv..

[20] Olsen SR (1954). Estimation of Available Phosphorus in Soils by Extraction with Sodium Bicarbonate.

[21] Murphy J, Riley JP (1962). A modified single solution method for the determination of phosphate in natural waters. Anal. Chim. Acta.

[22] Sanderman J, Amundson R, Holland HD, Turekian KK (2003). 8.07 - Biogeochemistry of Decomposition and Detrital Processing. Treatise on Geochemistry.

[23] Tavares RLM, Nahas E (2014). Humic fractions of forest, pasture and maize crop soils resulting from microbial activity. Braz. J. Microbiol..

[24] Francaviglia R, Renzi G, Ledda L, Benedetti A (2017). Organic carbon pools and soil biological fertility are affected by land use intensity in Mediterranean ecosystems of Sardinia, Italy. Sci. Total Environ..

[25] Kawahigashi M, Sumida H, Yamamoto K (2005). Size and shape of soil humic acids estimated by viscosity and molecular weight. J. Colloid Interface Sci..

[26] Culman, S. Soil Health and Active Organic Matter|Soil Fertility. https://soilfertility.osu.edu/extension-and-outreach/soil-health-testing (2020).

[27] Grubinger, V. Soil Organic Matter: The Living, the Dead, and the Very Dead. https://www.uvm.edu/vtvegandberry/factsheets/soilorganicmatter.html (2020).

[28] Larionova AA (2017). Effect of temperature and moisture on the mineralization and humification of leaf litter in a model incubation experiment. Euras. Soil Sc..

[29] Khademi Z, Jones DL, Malakouti MJ, Asadi F (2010). Organic acids differ in enhancing phosphorus uptake by *Triticum aestivum* L.—effects of rhizosphere concentration and counterion. Plant Soil.

[30] Mihoub A, Bouhoun MD, Naeem A, Saker ML (2016). Low-molecular weight organic acids improve plant availability of phosphorus in different textured calcareous soils. Arch. Agron. Soil Sci..

[31] Fink JR (2016). Iron oxides and organic matter on soil phosphorus availability. Ciênc Agrotec.

[32] Liu D (2008). Comparison of synthetic chelators and low molecular weight organic acids in enhancing phytoextraction of heavy metals by two ecotypes of *Sedum alfredii* Hance. J. Hazard. Mater..

[33] Yaping, M. A., Cao, B., Wang, Y., Agriculture, S. O. & University, N. Correlation analysis between soil nutrients and fruit quality in *Ziziphus jujuba* Lingwuchangzao. Nonwood Forest Research (2017).

[34] Mauromicale G, Longo AMG, Monaco AL (2011). The effect of organic supplementation of solarized soil on the quality of tomato fruit. Sci. Hortic..

[35] Fallahi B (2006). Influence of humic substances and nitrogen on yield, fruit quality, and leaf mineral elements of ‘early spur rome’ apple. J. Plant Nutr..

[36] Boondum S (2019). Carbon dioxide (CO_2_) enrichment in greenhouse enhanced growth and productivity of tomato (*Solanum lycopersicum* L.) during winter. Acta Hortic..

[37] Pan T (2019). Interaction of supplementary light and CO2 enrichment improves growth, photosynthesis, yield, and quality of tomato in autumn through spring greenhouse production. HortScience.

[38] Sulmon, C., Gouesbet, G., Couee, I. & El Amrani, A. *Method for improving the phytoremediation of polluted sites by providing plants with exogenous carbohydrates*. (US, 2012).

[39] Hirner A (2006). Arabidopsis LHT1 is a high-affinity transporter for cellular amino acid uptake in both root epidermis and leaf mesophyll. Plant Cell.

[40] Jones DL, Healey JR, Willett VB, Farrar JF, Hodge A (2005). Dissolved organic nitrogen uptake by plants–an important N uptake pathway?. Soil Biol. Biochem..

[41] Bhattacharyya P, Das S, Adhya TK (2013). Root exudates of rice cultivars affect rhizospheric phosphorus dynamics in soils with different phosphorus statuses. Commun. Soil Sci. Plant Anal..

[42] Breen S, Solomon PS, Bedon F, Vincent D (2015). Surveying the potential of secreted antimicrobial peptides to enhance plant disease resistance. Front. Plant Sci..

