# Supplementary material for: Effects of organic fertilizers via quick artificial decomposition on crop growth
Source: Sci Rep. 2021 Feb 16;11:3900. doi: 10.1038/s41598-021-83576-4 (PMC7887227; doi:10.1038/s41598-021-83576-4)
Supplement: Supplementary file 1 — Supplementary Information. [file 41598_2021_83576_MOESM1_ESM.docx]

**Effects of organic fertilizers via quick artificial decomposition on crop growth**

Xuemiao Ma^1^, Haixiao Li^2, 3*^, Yan Xu^4^, Cunshou Liu^1*^

^1^*College of Natural Resources and Environment, Northwest A&F University, Yangling, Shaanxi, 712100 China*

^2^*Nankai University, College of Environment Science and Engineering, Tianjin, 300350, China*

^3^*Tianjin Key Laboratory of Environmental Technology for Complex Trans-media Pollution, Tianjin, 300350 China*

^4^*Department of Soils and Agri-Food Engineering, Paul Comtois Bldg., Laval University, Quebec, QC, Canada G1K 7P4*

Co-corresponding authors:

**Cunshou Liu**

E-mail: Liucunshou@163.com

Telephone: 86-29-87080029;

Fax number: 86-29-87080029.

**Haixiao Li**

E-mail: lihaixiao001@gmail.com

Telephone: 86-13667138873;

Fax number:

**Number of tables**: 3

**Number of figures**: 2

**Supplementary annexes**

| **Table A.1** Species name, molecular mass and chemical formula of organic matters detected in the produced fertilizers | | | |
| --- | --- | --- | --- |
| **No.** | **Name** | **Molecular Mass** | **Formula** |
| 1 | (1S,2S)-3-oxo-2-PeNtyl-cycloPeNtaNebutaNoic acid | 240.17240.17 | C_14_ H_24_ O_3_ |
| 2 | (6R)- vitamin D2 6,19-sulfur dioxide adduct /  (6R)-ergocalciferol 6,19-sulfur dioxide adduct | 460.30460.30 | C_28_ H_44_ O_3_ S |
| 3 | (6RS)-22-oxo-23,24,25,26,27-PeNtaNorvitamiN  D3 6,19-sulfur dioxide adduct /  (6RS)-22-oxo-23,24,25,26 | 392.20392.20 | C_22_ H_32_ O_4_ S |
| 4 | 10-hydroxy-8E-DeceNe-2,4,6-triyNoic acid | 176.05176.05 | C_10_ H_8_ O_3_ |
| 5 | 11-(3-acetoxy-1-ProPyNyl)-1alPha,25-dihydroxy-9,11-didehydrovitamiN D3 / 11-(3-acetoxy-1-ProPyNyl)-1 | 510240.17.33 | C_32_ H_46_ O_5_ |
| 6 | 12-oxo-_14_,18-dihydroxy-9Z,13E,15Z-octadecatrieNoic acid | 324.19460.30 | C_18_ H_28_ O_5_ |
| 7 | 13-HydroxyPergolide | 330.18392.20 | C_19_ H_26_ N_2_ O S |
| 8 | 1-AmiNocycloProPaNe-1-carboxylic acid | 101.05240.17 | C_4_ H_7_ N O_2_ |
| 9 | 1H-INdole-3-acetic acid, 5-[[(methylamino) sulfonyl] methyl]- glucuronide | 458.10460.30 | C_18_ H_22_ N_2_ O_10_ S |
| 10 | 1-Hydroxy-2-NaPhthoic acid | 188.05392.20 | C_11_ H_8_ O_3_ |
| 11 | 1-ProPaNamiNe, N,N-dimethyl-3-(5-oxidodibeNzo[b,e]thiePiN-11(6H)-ylideNe)-, (E)- (9CI) | 311.13176.05 | C_19_ H_21_ N O S |
| 12 | 2,4,6-TrimethylacetoPheNoNe imine | 161.12240.17 | C_11_ H_15_ N |
| 13 | 2,4-DihydroxytacriNe | 230.11460.30 | C_13_ H_14_ N_2_ O_2_ |
| 14 | 2,6-PiPeridiNedicarboxylic acid | 173.07392.20 | C_7_ H_11_ N O_4_ |
| 15 | 2-amiNo-4'-hydroxy-ProPioPheNoNe | 165.08176.05 | C_9_ H_11_ N O_2_ |
| 16 | 2-AmiNoadiPic acid | 161.07392.20 | C_6_ H_11_ N O_4_ |
| 17 | 2beta,3alPha,7alPha,12alPha-Tetrahydroxy-5beta-cholaN-24-oic Acid | 424.28176.05 | C_24_ H_4_0 O_6_ |
| 18 | 2E,4E-hexadecadieNoic acid | 252.21 | C_16_ H_28_ O_2_ |
| 19 | 2H-INdol-2-oNe, 1,3-dihydro-4-[2-hydroxy-3-[(1-methylethyl)amino] Propoxy]- | 264.15 | C_14_ H_2_0 N_2_ O_3_ |
| 20 | 2-HydroxyadiPic acid | 162.05 | C_6_ H_10_ O_5_ |
| 21 | 2-Ketoglutaric Acid | 146.02 | C_5_ H_6_ O_5_ |
| 22 | 2-MethyliNdoliNe | 133.09 | C_9_ H_11_ N |
| 23 | 2-NaPhthylalaNiNe | 215.09 | C_13_ H_13_ N O_2_ |
| 24 | 2-oxoisocaProic acid | 130.06 | C_6_ H_10_ O_3_ |
| 25 | 2-PheNyl-1,3-ProPaNediyl monocarbamate | 195.09 | C_10_ H_13_ N O_3_ |
| 26 | 2-PheNylacetamide | 136.10 | C_8_ H_9_ N O |
| 27 | 2-Pyridylacetic acid | 137.05 | C_7_ H_7_ N O_2_ |
| 28 | 2R-amiNohePtaNoic acid | 145.11 | C_7_ H_15_ N O_2_ |
| 29 | 2-phenyl-1,3-propanediol monocarbamate | 195.09 | C_10_ H_13_ N O_3_ |
| 30 | 2-ketoglutaric acid | 146.02 | C_5_ H_6_ O_5_ |
| 31 | 3-(4-HydroxyPheNyl)Propionic acid | 166.06 | C_9_ H_10_ O_3_ |
| 32 | 3,5-hexadieNoic acid | 112.05 | C_6_ H_8_ O_2_ |
| 33 | 3 ', 5'-cyclic inosine monophosphate | 329.00 | C_10_ H_11_ N_4_ O_7_ P |
| 34 | 3-Deoxy-D-glycero-D-galacto-2-NoNulosoNic acid | 268.08 | C_9_ H_16_ O_9_ |
| 35 | 3-HydroxydodecaNedioic acid | 246.15 | C_12_ H_22_ O_5_ |
| 36 | 3-HydroxyPheNylacetate | 152.05 | C_8_ H_8_ O_3_ |
| 37 | 3-hydroxy-PheNylglycol | 154.06 | C_8_ H_10_ O_3_ |
| 38 | 3'-HydroxytrimethoPrim | 276.12 | C_13_ H_16_ N_4_ O_3_ |
| 39 | 3-keto Palmitic acid | 270.22 | C_16_ H_3_0 O_3_ |
| 40 | 3-Ketolactose | 340.10 | C_12_ H_2_0 O_11_ |
| 41 | 3-octeNal | 126.10 | C_8_ H_14_ O |
| 42 | 4-(diaminomethylideneamino) butanoic acid | 145.09 | C_5_ H_11_ N_3_ O_2_ |
| 43 | 4,12-dihydroxy-hexadecaNoic acid | 288.23 | C_16_ H_32_ O_4_ |
| 44 | 4,6,11-hexadecatrieNal | 234.20 | C_16_ H_26_ O |
| 45 | 4-AmiNo-4-deoxychorismic acid | 226.10 | C_10_ H_11_ N O_5_ |
| 46 | 4-hePtyNoic acid | 126.07 | C_7_ H_10_ O_2_ |
| 47 | 4-HydroxyPyridiNe | 96.00 | C_5_ H_5_ N O |
| 48 | 4-methylumbelliferyl β-D glucuronide | 351.10 | C_16_ H_16_ O_9_ |
| 49 | 4-hydroxy-3-p-methoxymandelic acid | 198.05 | C_9_ H_10_ O_5_ |
| 50 | 4-hydroxy-3-methoxymandelic acid | 198.05 | C_9_ H_10_ O_5_ |
| 51 | 5-(3,4-Dihydroxy-1,5-cyclohexadieN-1-yl)-5-PheNylhydaNtoiN | 286.10 | C_15_ H_14_ N_2_ O_4_ |
| 52 | 5-(4-Hydroxy-3-methoxyPheNyl)-5-PheNylhydaNtoiN | 298.10 | C_16_ H_14_ N_2_ O_4_ |
| 53 | 5-AmiNoPeNtaNoic acid | 117.08 | C_5_ H_11_ N O_2_ |
| 54 | 5-MethoxytryPtamiNe | 190.11 | C_11_ H_14_ N_2_ O |
| 55 | 6,19-ePidioxy-26,26,26,27,27,27-hexafluoro-25-hydroxy-6,19-dihydrovitamiN D3 / 6,19-ePidioxy-26,26,2 | 540.27 | C_27_ H_38_ F_6_ O_4_ |
| 56 | 6-hydroxy-7E,9E-OctadecadieNe-11,13,15,17-tetrayNoic acid | 280.11 | C_18_ H_16_ O_3_ |
| 57 | 6-HydroxyNicotiNe (R) | 178.11 | C_10_ H_14_ N_2_ O |
| 58 | 6-Ketoestriol | 302.15 | C_18_ H_22_ O_4_ |
| 59 | 7,8-DidehydroastaxaNthiN | 594.37 | C_4_0 H_5_0 O_4_ |
| 60 | 7-Deoxy-13-dihydroadriamyciNoNe | 400.12 | C_21_ H_2_0 O_8_ |
| 61 | 8,9,16-trihydroxyhexadecanoic acid | 304.23 | C_16_ H_32_ O_5_ |
| 62 | 9,12,13-trihydroxy-10-octadecenoic acid | 330.24 | C_18_ H_34_ O_5_ |
| 63 | 9-lauroleic acid | 198.16 | C_12_ H_22_ O_2_ |
| 64 | a,b-Dihydroxyisobutyric acid | 119.00 | C_4_ H_8_ O_4_ |
| 65 | Acamprosate | 181.04 | C_5_ H_11_ N O_4_ S |
| 66 | Adenine | 136.10 | C_5_ H_5_ N_5_ |
| 67 | a-hydroxybutyrate | 104.05 | C_4_ H_8_ O_3_ |
| 68 | Ala Ile | 202.13 | C_9_ H_18_ N_2_ O_3_ |
| 69 | Ala Leu Ala | 273.17 | C_12_ H_23_ N_3_ O_4_ |
| 70 | Ala Pro | 186.10 | C_8_ H_14_ N_2_ O_3_ |
| 71 | aluminum acetate | 204.02 | C_6_ H_9_ Al O_6_ |
| 72 | Aminosalicylic acid | 153.04 | C_7_ H_7_ N O_3_ |
| 73 | Anandamide (20:2, N-6) | 351.31 | C_22_ H_41_ N O_2_ |
| 74 | Arg Ala Gin | 373.21 | C_14_ H_27_ N_7_ O_5_ |
| 75 | Arg Lys | 302.21 | C_12_ H_26_ N_6_ O_3_ |
| 76 | Arg Phe Gly | 378.20 | C_17_ H_26_ N_6_ O_4_ |
| 77 | Arg Ser Ser | 348.18 | C_12_ H_24_ N_6_ O_6_ |
| 78 | Arg Trp | 360.19 | C_17_ H_24_ N_6_ O_3_ |
| 79 | Arg Val Val | 372.25 | C_16_ H_32_ N_6_ O_4_ |
| 80 | Asn Ala Glu | 332.13 | C_12_ H_2_0 N_4_ O_7_ |
| 81 | Asn Gly Ala | 260.11 | C_9_ H_16_ N_4_ O_5_ |
| 82 | Asn Gly Gly | 246.10 | C_8_ H_14_ N_4_ O_5_ |
| 83 | Asn Tyr Gly | 352.14 | C_15_ H_2_0 N_4_ O_6_ |
| 84 | Asp Lys Glu | 390.18 | C_15_ H_26_ N_4_ O_8_ |
| 85 | Asp Tyr | 296.10 | C_13_ H_16_ N_2_ O_6_ |
| 86 | Asp Val Asn | 346.15 | C_13_ H_22_ N_4_ O_7_ |
| 87 | Aspartame | 294.12 | C_14_ H_18_ N_2_ O_5_ |
| 88 | a-methyl-3,4-di-p-hydroxybenzoic acid | 196.07 | C_10_ H_12_ O_4_ |
| 89 | a-methyl-3,4-dihydroxyphenylpropionic acid | 196.07 | C_10_ H_12_ O_4_ |
| 90 | Benperidol | 381.19 | C_22_ H_24_ F N_3_ O_2_ |
| 91 | Benzene methanol, 2-(2-amiNoProPoxy)-3-methy | 196.11 | C_11_ H_16_ O_3_ |
| 92 | Benzoic acid | 122.04 | C_7_ H_6_ O_2_ |
| 93 | Benzyl benzoate | 212.08 | C_14_ H_12_ O_2_ |
| 94 | Benzyl malic acid | 224.07 | C_11_ H_12_ O_5_ |
| 95 | beta-vinyl acrylic acid | 98.04 | C_5_ H_6_ O_2_ |
| 96 | bis(4-fluoroPheNyl)-Methanone | 218.05 | C_13_ H_8_ F_2_ O |
| 97 | Bisacodyl | 361.13 | C_22_ H_19_ N O_4_ |
| 98 | bisdeallyalmitrine | 397.18 | C_2_0 H_21_ F_2_ N_7_ |
| 99 | Brompheniramine | 318.07 | C_16_ H_19_ Br N_2_ |
| 100 | butalbital | 224.12 | C_11_ H_16_ N_2_ O_3_ |
| 101 | C16 Sphinganine | 273.27 | C_16_ H_35_ N O_2_ |
| 102 | C24: 1-OH thioester | 905.63 | C_48_ H_91_ N O_12_ S |
| 103 | Carbaryl | 201.08 | C_12_ H_11_ N O_2_ |
| 104 | carbinoxamine | 290.12 | C_16_ H_19_ Cl N_2_ O |
| 105 | Carboxyltolmetin | 287.08 | C_15_ H_13_ N O_5_ |
| 106 | Carboxyprimaquine | 274.13 | C_15_ H_18_ N_2_ O_3_ |
| 107 | Carnosine | 227.10 | C_9_ H_14_ N_4_ O_3_ |
| 108 | Cerp(d18:1/26:0) | 783.65 | C_46_ H_9_0 N O_6_ P |
| 109 | Clonidine Metabolite 3 | 227.00 | C_9_ H_7_ Cl_2_ N_3_ |
| 110 | cortisone acetate | 402.20 | C_23_ H_3_0 O_6_ |
| 111 | Cys Cys Pro | 321.08 | C_11_ H_19_ N_3_ O_4_ S_2_ |
| 112 | Cys Gln Met | 380.12 | C_13_ H_24_ N_4_ O_5_ S_2_ |
| 113 | Cys Lys Met | 380.16 | C_14_ H_28_ N_4_ O_4_ S_2_ |
| 114 | Cys Val Phe | 367.16 | C_17_ H_25_ N_3_ O_4_ S |
| 115 | D-1-PiPerideiNe-2-carboxylic acid | 127.06 | C_6_ H_9_ N O_2_ |
| 116 | Dacarbazine | 182.09 | C_6_ H_10_ N_6_ O |
| 117 | Deoxyribose | 133.10 | C_5_ H_10_ O_4_ |
| 118 | desethyletomidate | 216.09 | C_12_ H_12_ N_2_ O_2_ |
| 119 | Demethylindomethacin | 343.06 | C_18_ H_14_ Cl N O_4_ |
| 120 | Dethiobiotin | 215.10 | C_10_ H_18_ N_2_ O_3_ |
| 121 | D-Glucose | 180.06 | C_6_ H_12_ O_6_ |
| 122 | Di-demethylcitalopram | 296.13 | C_18_ H_17_ F N_2_ O |
| 123 | Dihydromyricetin | 321.10 | C_15_ H_12_ O_8_ |
| 124 | Dihydroxyacetone (glycerone) | 89.00 | C_3_ H_6_ O_3_ |
| 125 | Dihydroxyphenylacetic acid | 168.04 | C_8_ H_8_ O_4_ |
| 126 | Diphenylmethylphosphine oxide | 216.07 | C_13_ H_13_ O P |
| 127 | ErgoliNe-8-methaNol, 10-methoxy-, (8b)- | 272.15 | C_16_ H_2_0 N_2_ O_2_ |
| 128 | ErgoliNe-8-methaNol, 10-methoxy-, (8b)- | 272.15 | C_16_ H_2_0 N_2_ O_2_ |
| 129 | Etomidate | 244.12 | C_14_ H_16_ N_2_ O_2_ |
| 130 | Flecainide | 414.14 | C_17_ H_2_0 F_6_ N_2_ O_3_ |
| 131 | Flecainide meta-O-dealkylated | 332.13 | C_15_ H_19_ F_3_ N_2_ O_3_ |
| 132 | Flecainide | 414.14 | C_17_ H_2_0 F_6_ N_2_ O_3_ |
| 133 | Fluocinolone acetonide | 452.20 | C_24_ H_3_0 F_2_ O_6_ |
| 134 | Fluocinonide | 494.21 | C_26_ H_32_ F_2_ O_7_ |
| 135 | flurandrenolide | 436.23 | C_24_ H_33_ F O_6_ |
| 136 | Flutamide | 276.07 | C_11_ H_11_ F_3_ N_2_ O_3_ |
| 137 | Fumaric acid | 115.00 | C_4_ H_4_ O_4_ |
| 138 | Gibberellin A8-catabolite | 362.14 | C_19_ H_22_ O_7_ |
| 139 | Glu Ala | 218.09 | C_8_ H_14_ N_2_ O_5_ |
| 140 | Glu Asp Ile | 375.16 | C_15_ H_25_ N_3_ O_8_ |
| 141 | Glu Glu | 276.10 | C_10_ H_16_ N_2_ O_7_ |
| 142 | Glu Glu Glu | 405.14 | C_15_ H_23_ N_3_ O_10_ |
| 143 | Glu Glu Ile | 389.18 | C_16_ H_27_ N_3_ O_8_ |
| 144 | Glu Ile Leu | 373.22 | C_17_ H_31_ N_3_ O_6_ |
| 145 | Glu Leu | 260.14 | C_11_ H_2_0 N_2_ O_5_ |
| 146 | Glu Leu Val | 359.21 | C_16_ H_29_ N_3_ O_6_ |
| 147 | Glu Pro | 244.11 | C_10_ H_16_ N_2_ O_5_ |
| 148 | Glu Ser Gln | 362.14 | C_13_ H_22_ N_4_ O_8_ |
| 149 | Glu Val Ala | 317.16 | C_13_ H_23_ N_3_ O_6_ |
| 150 | Glutaric acid | 131.00 | C_5_ H_8_ O_4_ |
| 151 | Gly Ala Gln | 274.13 | C_10_ H_18_ N_4_ O_5_ |
| 152 | Gly Asn Asp | 304.10 | C_10_ H_16_ N_4_ O_7_ |
| 153 | Gly Asn Pro | 286.13 | C_11_ H_18_ N_4_ O_5_ |
| 154 | Gly Gln Glu | 332.13 | C_12_ H_2_0 N_4_ O_7_ |
| 155 | Gly Gly Lys | 260.15 | C_10_ H_2_0 N_4_ O_4_ |
| 156 | Gly His | 212.09 | C_8_ H_12_ N_4_ O_3_ |
| 157 | Gly His Lys | 340.19 | C_14_ H_24_ N_6_ O_4_ |
| 158 | Gly Leu | 188.12 | C_8_ H_16_ N_2_ O_3_ |
| 159 | GPA(6:0/6:0) | 368.16 | C_15_ H_29_ O_8_ P |
| 160 | GPCho(O-18:0/20:4(5E,8E,11E,_14_E)) | 796.62 | C_46_ H_87_ N O_7_ P |
| 161 | Gpetn(18:0/20:4(5Z,8Z,11Z,_14_Z))[U] | 767.55 | C_43_ H_78_ N O_8_ P |
| 162 | Gpetnnme(O-16:0/O-16:0)[U] | 677.57 | C_38_ H_8_0 N O_6_ P |
| 163 | Guanidinosuccinic Acid | 176.10 | C_5_ H_9_ N_3_ O_4_ |
| 164 | Guanine | 151.05 | C_5_ H_5_ N_5_ O |
| 165 | Gulonolactone | 177.00 | C_6_ H_10_ O_6_ |
| 166 | Hexadecanedioic acid | 286.21 | C_16_ H_3_0 O_4_ |
| 167 | His Ala Asp | 341.13 | C_13_ H_19_ N_5_ O_6_ |
| 168 | His Glu | 284.11 | C_11_ H_16_ N_4_ O_5_ |
| 169 | His Leu | 268.15 | C_12_ H_2_0 N_4_ O_3_ |
| 170 | hydrocinnamic acid | 150.07 | C_9_ H_10_ O_2_ |
| 171 | Hydroxypentobarbital | 242.13 | C_11_ H_18_ N_2_ O_4_ |
| 172 | Hydroxyphenoxyethylaminohydroxypropanol | 227.12 | C_11_ H_17_ N O_4_ |
| 173 | Hypoxanthine | 137.00 | C_5_ H_4_ N_4_ O |
| 174 | Idebenone Metabolite (QS-4) | 268.09 | C_13_ H_16_ O_6_ |
| 175 | Idebenone Metabolite (QS-4) | 268.09 | C_13_ H_16_ O_6_ |
| 176 | Iduronic acid | 193.00 | C_6_ H_10_ O_7_ |
| 177 | Ile Ala Phe | 349.20 | C_18_ H_27_ N_3_ O_4_ |
| 178 | Ile Ala Val | 301.20 | C_14_ H_27_ N_3_ O_4_ |
| 179 | Ile Asn His | 382.20 | C_16_ H_26_ N_6_ O_5_ |
| 180 | Ile AsP | 246.12 | C_10_ H_18_ N_2_ O_5_ |
| 181 | Ile Glu Glu | 389.18 | C_16_ H_27_ N_3_ O_8_ |
| 182 | Ile Leu Leu | 357.26 | C_18_ H_35_ N_3_ O_4_ |
| 183 | Ile Val Val | 329.23 | C_16_ H_31_ N_3_ O_4_ |
| 184 | Indoleacetaldehyde | 159.07 | C_10_ H_9_ N O |
| 185 | Isoflurane | 183.97 | C_3_ H_2_ Cl F_5_ O |
| 186 | Isoquinoline n-oxide | 146.10 | C_9_ H_7_ N O |
| 187 | Isosorbide dinitrate | 236.03 | C_6_ H_8_ N_2_ O_8_ |
| 188 | Ketoprofen glucuronide | 430.13 | C_22_ H_22_ O_9_ |
| 189 | L-4-Hydroxy-3-methoxy-a-methylphenylalanine | 225.10 | C_11_ H_15_ N O_4_ |
| 190 | L-Cysteinylglycine disulfide | 297.05 | C_8_ H_15_ N_3_ O_5_ S_2_ |
| 191 | L-DOPA | 198.10 | C_9_ H_11_ N O_4_ |
| 192 | Leu Gly Val | 287.18 | C_13_ H_25_ N_3_ O_4_ |
| 193 | Leu Leu | 244.18 | C_12_ H_24_ N_2_ O_3_ |
| 194 | Leu Leu Ala | 315.22 | C_15_ H_29_ N_3_ O_4_ |
| 195 | Leu Leu Phe | 391.25 | C_21_ H_33_ N_3_ O_4_ |
| 196 | Leu Leu Pro | 341.23 | C_17_ H_31_ N_3_ O_4_ |
| 197 | Leu Phe Gly | 335.18 | C_17_ H_25_ N_3_ O_4_ |
| 198 | Leu Phe Pro | 375.22 | C_2_0 H_29_ N_3_ O_4_ |
| 199 | Leu Pro | 228.15 | C_11_ H_2_0 N_2_ O_3_ |
| 200 | Leu Trp | 317.17 | C_17_ H_23_ N_3_ O_3_ |
| 201 | Leu Val | 230.16 | C_11_ H_22_ N_2_ O_3_ |
| 202 | Leucine | 130.10 | C_6_ H_13_ N O_2_ |
| 203 | L-Formylkynurenine | 237.10 | C_11_ H_12_ N_2_ O_4_ |
| 204 | Lys Gln Ile | 387.25 | C_17_ H_33_ N_5_ O_5_ |
| 205 | Lys Ile | 259.19 | C_12_ H_25_ N_3_ O_3_ |
| 206 | malic acid | 134.02 | C_4_ H_6_ O_5_ |
| 207 | MalonylcarNitine | 229.10 | C_10_ H_15_ N O_5_ |
| 208 | Mesaconic acid | 130.03 | C_5_ H_6_ O_4_ |
| 209 | Met Asn Arg | 419.20 | C_15_ H_29_ N_7_ O_5_ S |
| 210 | Met Tyr | 312.11 | C_14_ H_2_0 N_2_ O_4_ S |
| 211 | Met Val | 248.12 | C_10_ H_2_0 N_2_ O_3_ S |
| 212 | Met Val Trp | 434.20 | C_21_ H_3_0 N_4_ O_4_ S |
| 213 | Methionine | 150.10 | C_5_ H_11_ N O_2_ S |
| 214 | Methoxyhydroxymethylhydrocinnamic acid | 210.09 | C_11_ H_14_ O_4_ |
| 215 | methyl 8-[2-(2-formyl-vinyl)-3-hydroxy-5-oxo-cyclopentyl]-octanoate | 310.18 | C_17_ H_26_ O_5_ |
| 216 | Methyl-3,4-dihydroxyphenylpropionic acid | 196.07 | C_10_ H_12_ O_4_ |
| 217 | Methylmalonic acid | 118.03 | C_4_ H_6_ O_4_ |
| 218 | Mexiletine | 179.13 | C_11_ H_17_ N O |
| 219 | Mitomycin | 334.13 | C_15_ H_18_ N_4_ O_5_ |
| 220 | N-(3S-hydroxy-butanoyl)-homoserine lactone | 187.08 | C_8_ H_13_ N O_4_ |
| 221 | N- (3-oxo-acetyl) -homoserine lactone | 213.10 | C_10_ H_15_ N O_4_ |
| 222 | N- (3-indoleacetyl) isoleucine | 288.15 | C_16_ H_2_0 N_2_ O_3_ |
| 223 | N,N-Didemethylchlorpromazine | 290.06 | C_15_ H_15_ Cl N_2_ S |
| 224 | N2,N2-Dimethylguanosine | 311.12 | C_12_ H_17_ N_5_ O_5_ |
| 225 | N2-Acetyl-L-orNithiNe | 175.10 | C_7_ H_14_ N_2_ O_3_ |
| 226 | N2-SucciNyl-L-orNithiNe | 232.11 | C_9_ H_16_ N_2_ O_5_ |
| 227 | N4-Acetylsulfamethoxazole | 295.06 | C_12_ H_13_ N_3_ O_4_ S |
| 228 | Nabumetone alcohol | 230.13 | C_15_ H_18_ O_2_ |
| 229 | N-Acetyl-P-benzoquinonimine | 149.05 | C_8_ H_7_ N O_2_ |
| 230 | N-Acetylserine | 147.05 | C_5_ H_9_ N O_4_ |
| 231 | N-Acetylserotonin | 218.11 | C_12_ H_14_ N_2_ O_2_ |
| 232 | N-Acrylylglycine methyl ester | 143.06 | C_6_ H_9_ N O_3_ |
| 233 | Nalbuphine-6-sulfate | 437.15 | C_21_ H_27_ N O_7_ S |
| 234 | N-Carboxyethyl-gamma-aminobutyric acid | 175.08 | C_7_ H_13_ N O_4_ |
| 235 | N-Carboxytocainide | 236.12 | C_12_ H_16_ N_2_ O_3_ |
| 236 | N-Carboxytocainide | 236.12 | C_12_ H_16_ N_2_ O_3_ |
| 237 | N-DealkylzucloPenthixol | 356.11 | C_2_0 H_21_ Cl N_2_ S |
| 238 | N-Desmethylclozapine | 312.11 | C_17_ H_17_ Cl N_4_ |
| 239 | Norharman | 169.10 | C_11_ H_8_ N_2_ |
| 240 | Norpromazine | 270.12 | C_16_ H_18_ N_2_ S |
| 241 | N-Seryltyrosine | 268.11 | C_12_ H_16_ N_2_ O_5_ |
| 242 | N-Succinyl-L-diaminopimelic acid | 290.11 | C_11_ H_18_ N_2_ O_7_ |
| 243 | N-acetylglucosamine 6-phosphate | 302.10 | C_8_ H_16_ N O_9_ P |
| 244 | Octulose-1,8-bisphosphate | 400.02 | C_8_ H_18_ O_14_ P_2_ |
| 245 | Oleoyl-CoA | 1031.36 | C_39_ H_68_ N_7_ O_17_ P_3_ S |
| 246 | Pantetheine | 278.13 | C_11_ H_22_ N_2_ O_4_ S |
| 247 | Pentoxifylline | 278.14 | C_13_ H_18_ N_4_ O_3_ |
| 248 | Pergolide sulfone | 346.17 | C_19_ H_26_ N_2_ O_2_ S |
| 249 | Phe Asp | 280.11 | C_13_ H_16_ N_2_ O_5_ |
| 250 | Phe Leu | 278.16 | C_15_ H_22_ N_2_ O_3_ |
| 251 | Phe Leu Asp | 393.19 | C_19_ H_27_ N_3_ O_6_ |
| 252 | Phe Phe | 312.15 | C_18_ H_2_0 N_2_ O_3_ |
| 253 | Phe Phe Leu | 425.23 | C_24_ H_31_ N_3_ O_4_ |
| 254 | Phe Pro Val | 361.20 | C_19_ H_27_ N_3_ O_4_ |
| 255 | Phe Tyr | 328.14 | C_18_ H_2_0 N_2_ O_4_ |
| 256 | Phe Tyr | 328.14 | C_18_ H_2_0 N_2_ O_4_ |
| 257 | Phe Val Val | 363.22 | C_19_ H_29_ N_3_ O_4_ |
| 258 | Phenacetine | 180.10 | C_10_ H_13_ N O_2_ |
| 259 | Phenylacetic acid | 136.05 | C_8_ H_8_ O_2_ |
| 260 | Phloionolic acid | 332.26 | C_18_ H_36_ O_5_ |
| 261 | Phloretin | 273.10 | C_15_ H_14_ O_5_ |
| 262 | P-Hydroxyaniline | 110.10 | C_6_ H_7_ N O |
| 263 | P-Hydroxyketorolac | 271.08 | C_15_ H_13_ N O_4_ |
| 264 | Phytosphingosine | 317.29 | C_18_ H_39_ N O_3_ |
| 265 | Pipecolic acid | 130.10 | C_6_ H_11_ N O_2_ |
| 266 | P-Methylolphenol | 124.05 | C_7_ H_8_ O_2_ |
| 267 | Porphobilinogen | 227.10 | C_10_ H_14_ N_2_ O_4_ |
| 268 | Pro Arg Pro | 368.22 | C_16_ H_28_ N_6_ O_4_ |
| 269 | Pro Asn Asp | 344.13 | C_13_ H_2_0 N_4_ O_7_ |
| 270 | Pro Gly Gln | 300.14 | C_12_ H_2_0 N_4_ O_5_ |
| 271 | Pro Phe | 262.13 | C_14_ H_18_ N_2_ O_3_ |
| 272 | Pro Pro Pro | 309.17 | C_15_ H_23_ N_3_ O_4_ |
| 273 | Pro Ser Glu | 331.14 | C_13_ H_21_ N_3_ O_7_ |
| 274 | Pro Tyr | 278.13 | C_14_ H_18_ N_2_ O_4_ |
| 275 | Pro Val Ile | 327.22 | C_16_ H_29_ N_3_ O_4_ |
| 276 | Pro Val Tyr | 377.20 | C_19_ H_27_ N_3_ O_5_ |
| 277 | Proline | 116.10 | C_5_ H_9_ N O_2_ |
| 278 | Promazine | 284.13 | C_17_ H_2_0 N_2_ S |
| 279 | Propanoic acid, 2-hydroxy-3-[(4-hydroxy-1-naphthalenyl) oxy]- | 248.07 | C_13_ H_12_ O_5_ |
| 280 | Propionylglycine methyl ester | 145.07 | C_6_ H_11_ N O_3_ |
| 281 | Protopine | 353.13 | C_2_0 H_19_ N O_5_ |
| 282 | Protoporphyrinogen IX | 568.31 | C_34_ H_4_0 N_4_ O_4_ |
| 283 | Pseudoephedrine | 165.12 | C_10_ H_15_ N O |
| 284 | Punaglandin 2 | 558.22 | C_27_ H_39_ Cl O_10_ |
| 285 | Purine | 120.04 | C_5_ H_4_ N_4_ |
| 286 | Pyroglutamic acid | 128.00 | C_5_ H_7_ N O_3_ |
| 287 | Ramiprilat | 388.20 | C_21_ H_28_ N_2_ O_5_ |
| 288 | Ribothymidine | 258.09 | C_10_ H_14_ N_2_ O_6_ |
| 289 | Ser Gly Arg | 318.17 | C_11_ H_22_ N_6_ O_5_ |
| 290 | Ser Pro | 202.10 | C_8_ H_14_ N_2_ O_4_ |
| 291 | Ser Pro Asn | 316.14 | C_12_ H_2_0 N_4_ O_6_ |
| 292 | Spectinomycin | 332.16 | C_14_ H_24_ N_2_ O_7_ |
| 293 | Sucrose | 342.12 | C_12_ H_22_ O_11_ |
| 294 | Sulfaphenazole | 313.10 | C_15_ H_14_ N_4_ O_2_ S |
| 295 | Terbutaline-1-glucuronide | 401.17 | C_18_ H_27_ N O_9_ |
| 296 | Tetracaine N-oxide | 280.18 | C_15_ H_24_ N_2_ O_3_ |
| 297 | Tetracaine | 264.18 | C_15_ H_24_ N_2_ O_2_ |
| 298 | Thr Gln Ser | 334.15 | C_12_ H_22_ N_4_ O_7_ |
| 299 | Thr Met | 250.10 | C_9_ H_18_ N_2_ O_4_ S |
| 300 | Thr Ser Arg | 362.19 | C_13_ H_26_ N_6_ O_6_ |
| 301 | Thr Ser Phe | 353.16 | C_16_ H_23_ N_3_ O_6_ |
| 302 | Thr Tyr | 282.12 | C_13_ H_18_ N_2_ O_5_ |
| 303 | Thromboxane A2 | 352.23 | C_2_0 H_32_ O_5_ |
| 304 | TraNs-4-Hydroxy-L-Proline | 131.06 | C_5_ H_9_ N O_3_ |
| 305 | Trifluoromethylphenylpropanediol | 220.07 | C_10_ H_11_ F_3_ O_2_ |
| 306 | Triparanol | 143.09 | C_7_ H_13_ N O_2_ |
| 307 | Trp Gly Asn | 375.15 | C_17_ H_21_ N_5_ O_5_ |
| 308 | Trp Pro | 301.14 | C_16_ H_19_ N_3_ O_3_ |
| 309 | Trp Trp Gln | 518.23 | C_27_ H_3_0 N_6_ O_5_ |
| 310 | Tyr Ala | 252.11 | C_12_ H_16_ N_2_ O_4_ |
| 311 | Tyr Glu | 310.12 | C_14_ H_18_ N_2_ O_6_ |
| 312 | Tyr Ile | 294.16 | C_15_ H_22_ N_2_ O_4_ |
| 313 | Tyr Ile Phe | 441.23 | C_24_ H_31_ N_3_ O_5_ |
| 314 | Tyr Tyr | 344.14 | C_18_ H_2_0 N_2_ O_5_ |
| 315 | Tyr Val | 280.14 | C_14_ H_2_0 N_2_ O_4_ |
| 316 | Tyr Val Glu | 409.18 | C_19_ H_27_ N_3_ O_7_ |
| 317 | Tyr Val Phe | 427.21 | C_23_ H_29_ N_3_ O_5_ |
| 318 | UDP-2,3- Diacyl-glucose | 1017.46 | C_43_ H_77_ N_3_ O_2_0 P_2_ |
| 319 | Val Asp Phe | 379.17 | C_18_ H_25_ N_3_ O_6_ |
| 320 | Val Ile Leu | 343.25 | C_17_ H_33_ N_3_ O_4_ |
| 321 | Val Leu Phe | 377.23 | C_2_0 H_31_ N_3_ O_4_ |
| 322 | Val Ser Arg | 360.21 | C_14_ H_28_ N_6_ O_5_ |
| 323 | Val Trp | 303.16 | C_16_ H_21_ N_3_ O_3_ |
| 324 | Val Val | 216.15 | C_10_ H_2_0 N_2_ O_3_ |
| 325 | Val Val Asp | 331.17 | C_14_ H_25_ N_3_ O_6_ |
| 326 | γ-hydroxyphenylbutazone glucuronide | 500.18 | C_25_ H_28_ N_2_ O_9_ |
| 327 | Iduronic acid | 194.17 | C_6_ H_10_ O_7_ |
| 328 | Ribulose | 150.05 | C_5_ H_10_ O_5_ |
| 329 | Trimethoprim 1-n-Methoxyzine | 306.13 | C_14_ H_18_ N_4_ O_4_ |
| 330 | Methoxyestradiol | 382.15 | C_19_ H_26_ O_6_ S |
| 331 | Tyrosine | 182.10 | C_9_ H_11_ N O_3_ |
| 332 | Deethylbenzimidate | 216.09 | C_12_ H_12_ N_2_ O_2_ |
| 333 | Devinyl-N-acetyl norfloxacin | 335.13 | C_16_ H_18_ F N_3_ O_4_ |
| 334 | Triaminoguanidine hydrochloride | 606.80 | C_14_ H_12_ I_3_ N O_2_ |
| 335 | Salicylic acid | 138.03 | C_7_ H_6_ O_3_ |
| 336 | Cis-3- (6-hydroxy-7-methoxy-5-benzofuranyl) acrylic acid glucuronide | 410.08 | C_18_ H_18_ O_11_ |
| 337 | Tylosin | 915.52 | C_46_ H_77_ N O_17_ |
| 338 | Vitamin C | 176.03 | C_6_ H_8_ O_6_ |
| 339 | Citalopram | 325.11 | C_19_ H_16_ F N O_3_ |
| 340 | Allyl-N- [3- (dimethylamino) propyl] -N- (ethylcarbamoyl) ergoline-8-formamide | 330.18 | C_19_ H_26_ N_2_ O S |
| 341 | Sulfinyl indenacylsulfide glucuronide | 516.13 | C_26_ H_25_ F O_8_ S |
